# Facile Electret Fabrication for Energy Harvesting from Human Gait

**DOI:** 10.3390/polym17050664

**Published:** 2025-02-28

**Authors:** Michele Zanoletti, Paolo Vitulo, Riccardo Morina, Daniele Callegari, Riccardo Viola, Piercarlo Mustarelli, Maddalena Patrini

**Affiliations:** 1 Dipartimento di Fisica and Centro di Ricerca Interdipartimentale in Materiali Avanzati e Dispositivi (MADE), Università degli Studi di Pavia, Via A. Bassi 6, 27100 Pavia, Italy; paolo.vitulo@unipv.it (P.V.); maddalena.patrini@unipv.it (M.P.); 2 Istituto Nazionale di Fisica Nucleare-Pavia, Via Bassi 6, 27100 Pavia, Italy; 3 Dipartimento di Scienza dei Materiali, Università degli Studi di Milano Bicocca, Via R. Cozzi 55, 20125 Milano, Italy; r.morina@campus.unimib.it (R.M.); piercarlo.mustarelli@unimib.it (P.M.); 4 Dipartimento di Chimica, Università degli Studi di Pavia, Via T. Taramelli 12, 27100 Pavia, Italy; daniele.callegari01@universitadipavia.it; 5 Atom S.p.A., Via Morosini 6, 27029 Vigevano, Italy; riccardo.viola@atom.it

**Keywords:** electret, corona charging, dielectric elastomer generators, energy harvesting, human gait

## Abstract

Dielectric elastomer generators (DEGs) are electrostatic transducers capable of harvesting electrical energy from oscillating mechanical parts and storing it in a battery or supercapacitor. The energy conversion element typically consists of a flexible capacitor with a variable capacitance that depends on the applied stress cycle and requires an external voltage source (bias voltage). In designing an energy harvesting device from human gait, we propose integrating two components: a dielectric elastomer fabricated using a nanocomposite polyurethane (TPU-CaCu_3_Ti_4_O_12_) and an electret serving as a bias voltage source. In this work, we report on the electret fabrication and long-term charge retention properties using corona charging. The manufactured electrets are tested in coupling with the dielectric elastomer and allowed us to harvest an energy amount of 62 µJ/cycle (3.1 µJ/cm^2^) on a resistive load of 450 MΩ during motion cycles at a frequency of 0.5 Hz. Given the materials used, this approach is well suited to harvesting energy from human gait and holds promise for powering wearable devices.

## 1. Introduction

The quest for energy harvesting (EH) from the environment is currently a hot topic, with so many dissimilar sources whose energy is wasted instead. Harvesting systems capable of transforming mechanical vibration into electrical energy have indeed attracted considerable interest throughout the last decade [1]. Among the various sources of readily available energy, human gait is very appealing for its simplicity [2]. As for the physical effects and materials involved, most of the research activities refer to classical piezoelectric ceramics, but triboelectric sources embedded in polymer elastomeric matrices are now increasingly considered. Traditional piezoelectric materials are rigid and difficult to incorporate without compromising flexibility. Our group has instead proposed an electrostrictive material based on a commercial thermoplastic polyurethane (TPU) elastomer, which is a material class widely used in the shoe industry [3]; it is soft, flexible, and can be seamlessly integrated into the shoe sole without disrupting comfort or gait. These DEG materials represent the basic components of harvesting platforms, which are adopted for powering wearable electronics in the Internet of Things (IoT) world. Moreover, most energy harvesters need a bias voltage to operate. In our research project, we investigate an electret as the bias voltage source of an electrostrictive system, being compatible with a shoe sole structure and capable of recovering energy from the human gait. (The description of the specific device intended to harvest energy from human gait is beyond the scope of the present manuscript. However, the reader interested in the energy-harvesting concept of a shoe can refer to the following link to find Supplementary Materials https://fitnessmaterialiavanzati2019.wordpress.com/news, accessed on 30 January 2025).

Electrets are traditionally considered the electrical counterpart of ferromagnetic materials for magnetism [4]; they are materials with permanent electrical polarization and should be used as electrical field generators. In recent years, they have gained renewed interest in replacing the voltage bias generator as a source of polarization for DEGs. Jean-Mistral et al. [5] introduced the combination of electrets with soft energy harvesters; they coupled the electret to the DEG in such a way as to create a current flow of opposite signs each time the air gap in between opens or closes. In this system, the electrets were manufactured by the corona discharge method [6], obtaining a surface voltage value of −1 kV and surface charge density σ_S_ = −800 nC/cm^2^. This system was able to scavenge 33 µJ/cycle. By the same method, Lee et al. [7] were able to charge a polytetrafluoroethylene (PTFE) electret to a surface potential of 1 kV (σ_S_ = 190 nC/cm^2^), while Genter et al. [8] obtained a parylene-C electret with a surface voltage of −600 V. A flexible electret membrane consisting of a sandwich configuration of PTFE and Tetrafluorethylene-hexafluorpropylene vinylidenfluoride (THV) with the pattern PTFE/THV/PTFE was realized by Huiming et al. [9]. This electret, polarized by thermal charging, exhibits a surface potential of −550 V (σ_S_ = −14.3 mC/cm^2^) and shows distinctive temperature stability and outstanding resilience to harsh environments and stress fatigue. The harvester assembled with this membrane reaches a power output of 4.66 µW. Huayang Li et al. [10] applied corona charging to a bilayer of a PTFE film and PTFE-polydimethysiloxane (PDMS) nanocomposite obtaining an electret with a surface voltage of −2.4 kV (σ_S_ = −50 nC/cm^2^). This value was stably maintained with a decay of 2% after 25 days and showed better than 70% recovery after dipping in water. A peak power output of 54 mW was achieved. Peter et al. [11] reached to scavenge a 206 µJ/cycle with an electret-based converter. Sakane et al. [12] developed a novel electret material by introducing a silane-coupling reagent into the CYTOP^®^ perfluorinated polymer and charging it with corona charging, achieving a surface charge density of −1.5 mC/cm^2^ and power peaks of 0.7 mW. Regarding alternative energy harvesting technologies, piezoelectric materials such as lead zirconate titanate (PZT) exhibit good power density; however, they are brittle ceramics and not typical materials used in shoe soles. Their inherent rigidity would also introduce discomfort, making them less suitable for wearable applications. Similarly, triboelectric nanogenerators (TENGs) could be a viable option; however, their operation at high voltage and low current, along with potential limitations in energy and power density, may reduce their effectiveness for powering low-impedance portable electronics [13]. In contrast, our dielectric elastomer generator (DEG) system offers a more suitable solution, providing flexibility, comfort, and better adaptability for integration into footwear, while also delivering higher current density at lower voltage—key factors for efficiently powering wearable electronics [3]. In summary, the literature to date demonstrates how this technology can power electronic components at low consumption, being more sustainable than button cell batteries.

In this work, we produced corona-charged thin film electrets to supply a DEG system designed for a shoe sole with a long-lasting bias voltage. The harvester should sustain the energy consumption of a sensor platform inserted in the sole to monitor biophysical parameters. The idea is to create a device that could be embedded in the TPU of the sole; therefore, we focused on verifying that this environment is suitable for preventing electret discharge, which is one of its main challenges. For this reason, we conducted tests on the durability of the electret within the TPU to assess its long-term stability in these conditions. As for the electret material, we choose foils of Fluorinated Ethylene Propylene (FEP), a dielectric material that maintains a quasi-permanent electrical charge upon corona charging. FEP is well-established in the literature for its suitability in such applications [14], and it is also cost-effective and readily available. The shape and thickness of the FEP foil used for the electret were selected to achieve the best capacitive coupling and to ensure sufficient flexibility, allowing it to bend without issues. Beyond the surface voltage value, this configuration enabled the device to achieve good energy harvesting performance. In this work, we introduce the use of a TPU bridge, which ensures capacitive coupling and allows soft deformation that adapts to the shoe’s movement during walking. Additionally, we have charged the electret directly onto the TPU structure, observing that this configuration allows effective energy collection. This design overcomes the challenge of integrating energy harvesting technology into wearable devices by maintaining comfort and flexibility. The actual integration of the device into the footwear will be addressed in future work.

## 2. Materials and Methods

### 2.1. Materials and Devices

#### 2.1.1. Materials

The material used for the dielectric elastomer (DE) fabrication is TPU-CaCu_3_Ti_4_O_12_ (CCTO), with its overall physico-chemical characterization detailed in [3]. All other materials used in this work are commercially available. DuPont™ (Wilmington, DE, USA) Fluorinated Ethylene Propylene (FEP) foils, 50 µm in thickness, are employed in the electret fabrication. FEP is characterized by a dielectric strength of 70 MV/m and a dielectric constant (ε_r_) at 25 °C, 100 Hz–1 MHz of 2 [15]. The material utilized in the 3D printing process is Stratasys Fused Deposition Modeling (FDM) TPU 92A (Stratasys, MN, USA), with the datasheet provided in [16]. Additionally, a 1 mm thick Polyethylene Terephthalate (PET) cylinder is used during the corona charging process in the electret fabrication [17].

#### 2.1.2. Devices

The instruments and equipment used in the experiments and measurements are listed below, along with their main technical specifications. A CAEN A1526N Power Supply (CAEN, Viareggio, Italy) high-voltage generator, with a maximum output of −10 kV, was employed to provide the high voltage for the corona charging process. The surface voltage value of the electrets was measured using a hand-held electrostatic voltmeter, the AlphaLab Inc. SV2999 M2 (AlphaLab, Pittsburgh, PA, USA). The mechanical strain applied to the DE was facilitated by an automatic slide system, which consists of three devices: an electric slide (Festo-Esslingen, Germany-GSK-46-200-10P), a motor controller (Festo CMMP-AS-C2-3A-M0), and a servo motor (Festo EMME-AS-60-S). The voltmeter used for measurements was a Picoscope 2204Aoscilloscope (Picoscope, St Neots, United Knigdom). To ensure accurate voltage measurements, an isolation op-Amp (Analog Devices—Wilmington, MA, USA—AD202K) was used in combination with the Picoscope 2204A oscilloscope. The op-Amp features unitary gain and an input impedance of 10^12^ Ω. For current measurements, a Keithley 4517B pico-ammeter was utilized (Keithley Instruments, Solon, OH, USA). Lastly, the 3D printing was performed using the FDM F170 and F123 series 3D printers from Stratasys.

### 2.2. Electret Fabrication and Discharge Monitoring

**Figure 1:**
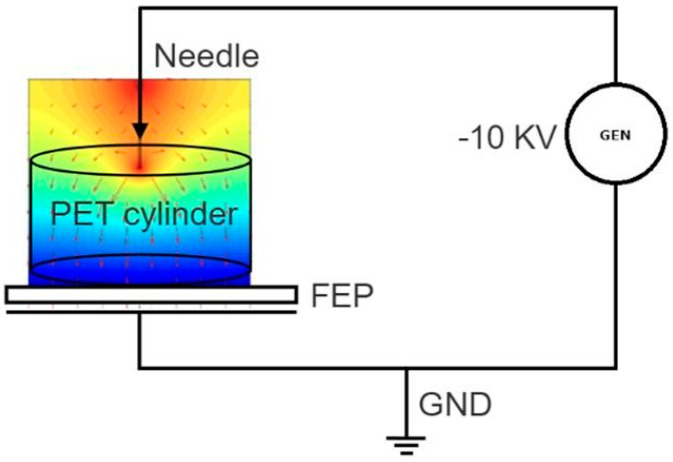
Schematic of the corona charging setup.

When designing an energy harvester for capturing energy from human motion, a fundamental aspect is to ensure the non-invasiveness to the natural walking movement [18]. For this reason, we created electrets of adequate size, thin, and flexible so that they can easily bent inside a shoe sole along the walking motion. We used the 50 µm FEP foils to fabricate electrets by the corona charging method [19]. The schematic of the experimental setup is reported in Figure 1. It consists of a high-voltage generator connected to a stainless-steel needle, with a holder to maintain the desired distance between the needle under HV and the FEP film surface to be charged. A ground copper electrode is added below the FEP to close the circuit. Additionally, a cylinder made of PET material was placed between the FEP surface and the tip of the needle.

By varying the distance and the HV value, it is possible to modulate the electric field, which must not exceed the breakdown field of FEP (about 70 MV/m). When the desired high voltage—in our case of a negative sign—is switched on, an ionization region is established in the air, where charged ions are produced. These ions are accelerated, and the negative ones are projected onto the surface of the sample. This mechanism results in the implantation of negative charges at the surface and into the volume of the FEP material.

Our aim is to use the electret as a bias voltage generator, then we are interested in the surface voltage, the surface charge density, and the discharge trend over time.

The surface area and shape of these electrets can be modulated as desired. Moreover, as referred to our typical electret size (10–20 cm^2^) the surface charge density could be tuned, both in positive and negative signs. This charge distribution produces the desired electric field at a variable distance in the range of a few millimeters (see below), which, joined to the mechanical flexibility of FEP, allows these electrets to be used as a polarization field source in the context of a TPU-based footwear sole.

The surface voltage of the electrets was measured with the hand-held electrostatic voltmeter. The relationship between the surface charge density (*σ_S_* [C/m^2^]), surface voltage (*V_S_* [V]), the FEP dielectric properties (*ε_0_* [F/m], *ε_r_* [-]), the total implanted charge (*Q* [C]) and the film geometric properties (Area *A* [m^2^], thickness *d* [m]) is

(1) $$\sigma_{S}=\frac{Q}{A}=\frac{\varepsilon_{r}*\varepsilon_{0}*Vs}{d},$$

The lifespan of an electret is directly related to the loss of its electric charge through physical processes dependent on several factors, including environmental or material-driven ones. In our case, we wanted to test the lifespan of an electret maintained in the air as compared to the one stored in a TPU case (made of Stratasys FDM TPU 92A), which is the material several shoe soles are made of. We fabricated several electrets with the previously described fabrication method starting from a 4 cm × 5 cm and 50 µm FEP foil.

### 2.3. Energy Harvesting Tests

#### 2.3.1. Working Principle

**Figure 2:**
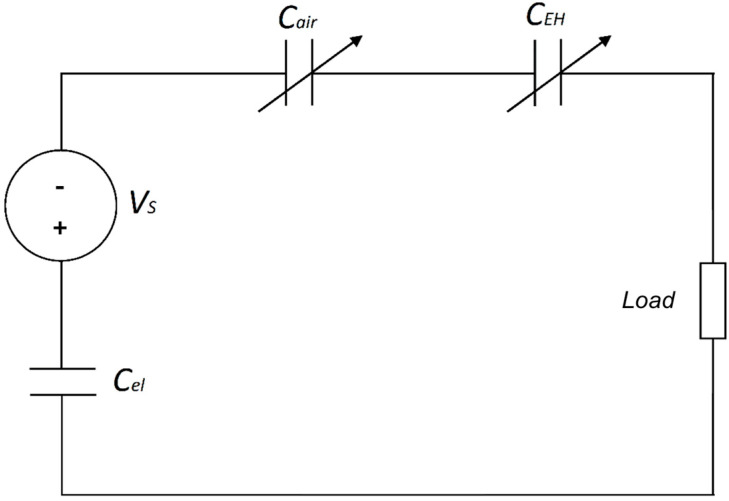
Equivalent circuit scheme of the energy harvesting setup.

As stated above, in this work, the electret is used as an internal bias voltage generator for energy harvesting tests, in the substitution of an external hard voltage generator. The conversion from mechanical to electrical energy is achieved by inducing a charge rearrangement between two electrodes at the ends of a load. An equivalent circuit scheme is reported in Figure 2, where the electret is schematized by the series of a voltage generator vs. a capacitor *C_el_*.

**Figure 3:**
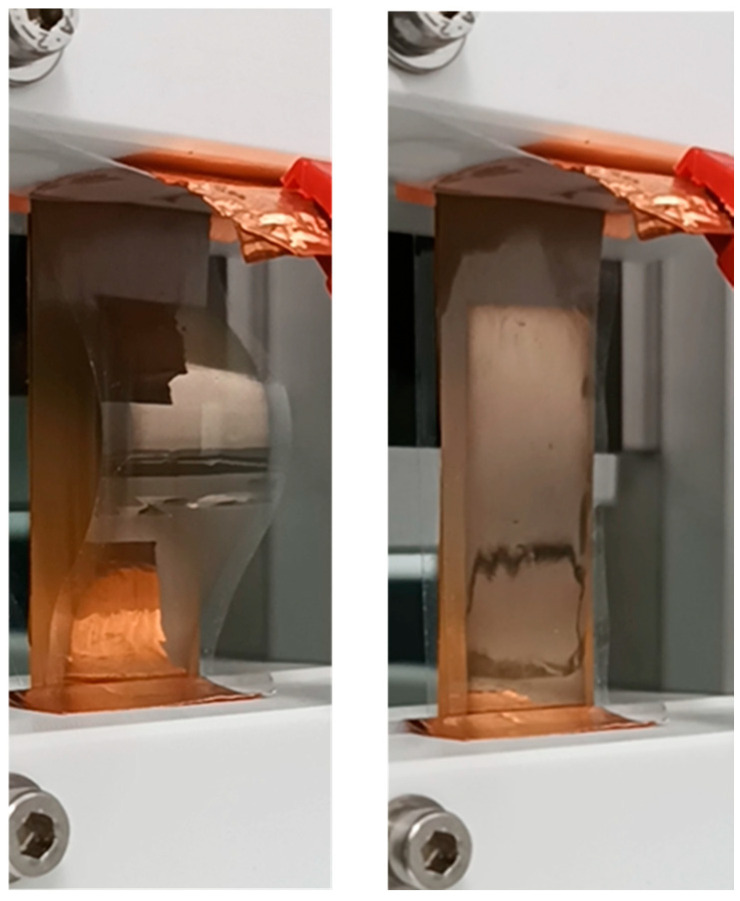
EH tests on resistive load.

**Figure A1:**
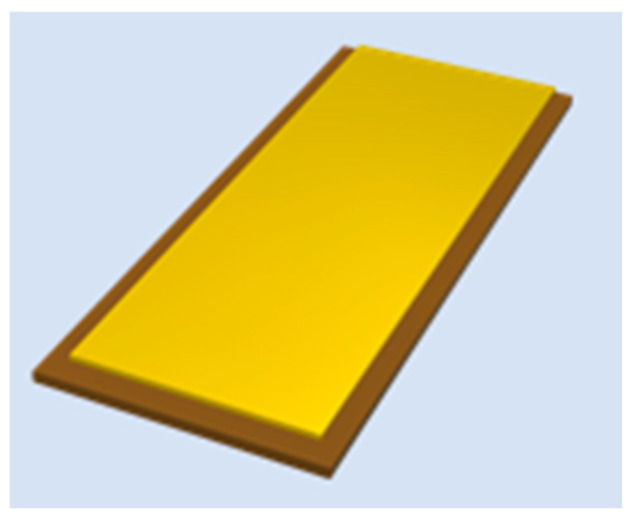
Au electrode deposited on the elastomer’s face with a size of 5.5 cm × 1.5 cm derived through a sputtering process. The Au sputtering process lasted approximately 1 min allowing the electrodes to reach a surface resistance of a few hundred Ohms.

To obtain the electrical charges’ rearrangement, the electret needs to be employed in a particular configuration for the energy harvesting operation cycle (about 1 Hz period), coupled to the dielectric elastomer. We chose as DE a home-made strip fabricated with a nanocomposite polyurethane (TPU-CaCu_3_Ti_4_O_12_ 50:50 *v*/*v*), as previously reported by our group [3]. In resting conditions, the electret and the DE are coupled so that the DE is fully stretched while the electret is curved away from it (e.g., see Figure 3). These coupled elements are connected to the load resistance *R_load_* with two electrodes. The first principal electrode is on the back surface of the electret. The electret’s charged surface, on the other hand, directly faces the bare side of the DE strip, which has a Au electrode layer on its opposite side towards the load (Figure A1).

The two variable capacitances are due to the air gap between the electret and DE strip and the DE strip elongation itself. The stretching of the dielectric element causes the electret to straighten until it is no longer bent, with its entire surface in contact with the electrodeless side of the dielectric. At the same time, the motion cycle itself increases and decreases the distance between the electret and the elastomer, thus changing the *C_air_* value. Then, both *C_air_* and *C_eh_* changes during this operation induce the variation in the capacitance of the overall system. The capacitance change induces a rearrangement of electrical charges between the two electrodes resulting in an electric current flowing through the load.

The total capacitance value *C_t_* [F] is given by the series of electret, air, and strip capacitance [6]:

(2) $$\frac{1}{C_{t}}=\frac{1}{C_{el}}+\frac{1}{C_{air}}+\frac{1}{C_{EH}},$$

where *C_el_* is the electret capacitance (given by $C_{el}=\frac{\varepsilon_{r}*\varepsilon_{0}*A}{d}$), while *C_air_*, and *C_EH_* are that of the air gap and the EH elastomer, respectively. The following equations describe the operation of the system [20] in the case the load is resistive (*R_load_* [Ω]). Based on Kirchhoff’s law and the differential equations governing the electrostatic system, the current intensity flowing through the load resistor can be expressed as

(3) $$i(t)=\frac{\partial Q}{\partial t}=\frac{V_{s}}{R_{load}}-\frac{Q}{R_{load}\;C_{t}},$$

where *Q* [C] is the charge induced on the DE electrode due to the charge of the electret surface, *V_s_* [V] is its surface voltage, and *R_load_* [Ω] is the load resistance value. The power dissipated (P [W]) on the load in a time interval (*t*_1_, *t*_2_) is given by

(4) $$P=\int_{t_{1}}^{t_{2}}R_{load}{\left(\frac{\partial Q}{\partial t}\right)}^{2}dt,$$

#### 2.3.2. Energy Harvesting Tests on a Resistive Load

**Figure A2:**
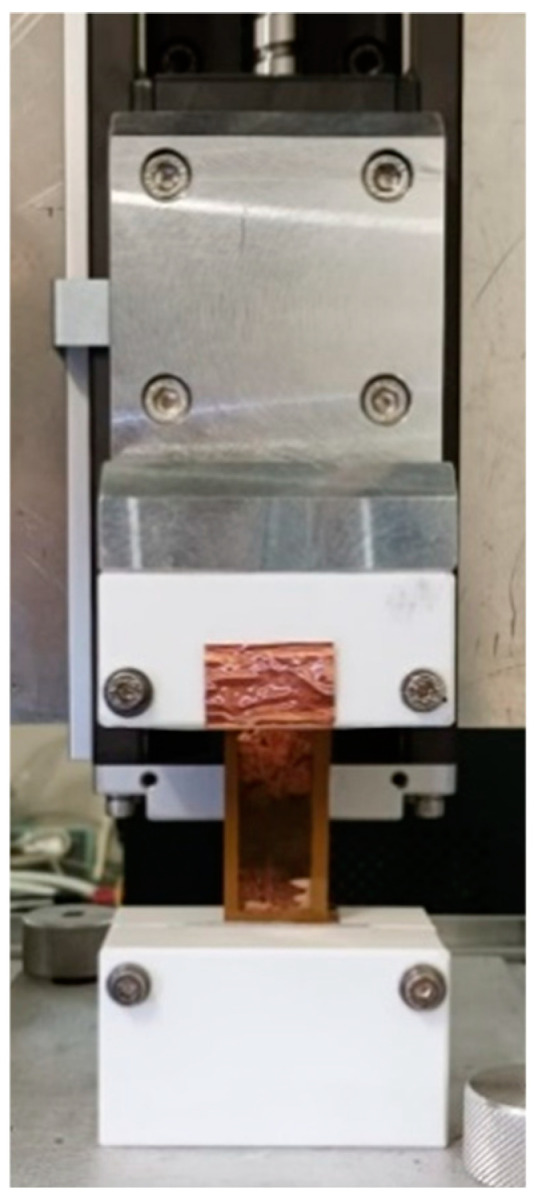
Picture of the Festo mechanical slide with clamps for EH tests.

We have set up a laboratory system for EH tests with different parts: mechanical motion, custom sample holder, voltage and current meters, and data acquisition devices. The Festo automated slide device has been used to perform mechanical stretching cycles. The system includes a precision electric slide (EGSK-46-200-10P), the motor controller (CMMP-AS-C2-3A-M0), and the servo motor (EMME-AS-60-S). On the slide, two customized clamps hold the samples to be strained/released, as illustrated in Figure 3 and Figure A2.

**Figure 4:**
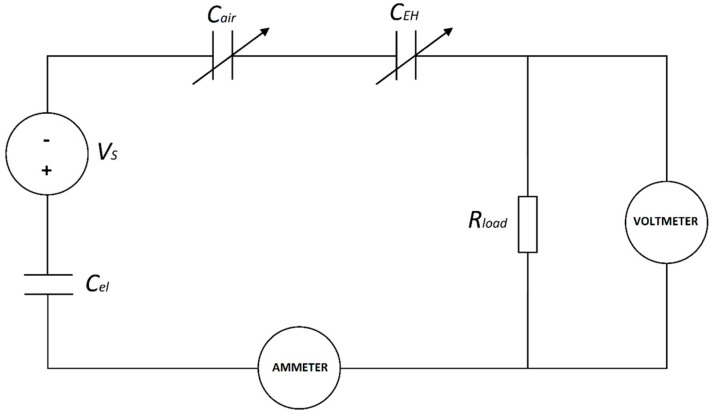
Equivalent circuit scheme of the electret coupling to the EH system with the measurement instruments adopted.

To measure the voltage value across the load we used the isolation op-Amp (Analog Devices AD202K) with unitary gain and an input impedance of 10^12^ Ω. The voltage signal was then visualized with the Picoscope 2204A oscilloscope (“Voltmeter” in the scheme), while the current intensity signal was acquired with the Keithley 4517B pico-ammeter. The equivalent electrical circuit scheme with the resistive load and the measurement instruments is reported in Figure 4.

During operation, the EH elastomer is subjected to stress cycles of 0.3 Hz (to simulate a low-frequency human gait), which changes its geometric dimension and consequently its capacitance value. The central part of the electret reaches a distance of 1 cm from the DE film. By repeating several cycles, one should calculate the energy per unit time which could be harvested from the motion and temporarily stored (e.g., in a battery or supercap). Due to a quasi-stationary working regime, the *V_s_* value should be assumed constant. In Table 1 we summarize the typical parameter values during the test.

#### 2.3.3. Energy Harvesting Tests in Optimized Configuration on a Resistive Load

**Figure 5:**
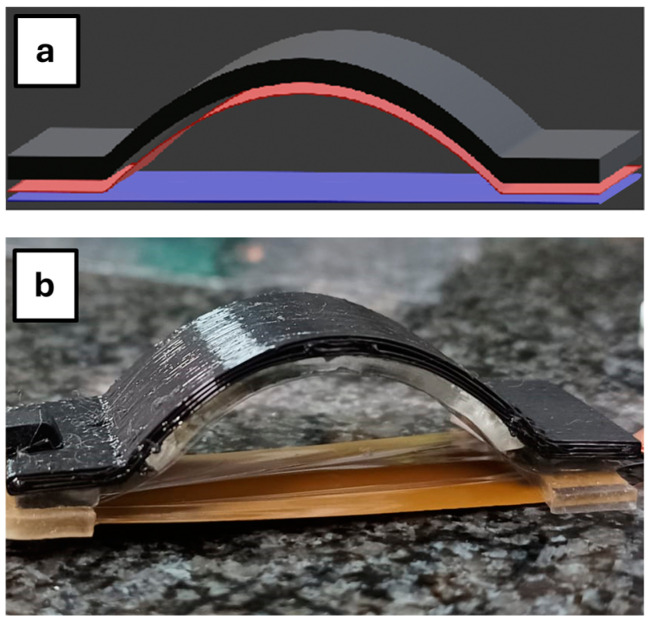
(**a**) Optimized architecture design. The black part represents the TPU printed structure, the red layer represents the FEP electret, and the blue layer represents the DE strip, (**b**) optimized architecture mockup.

Having established that it is possible to scavenge energy from the flexible electret foil, we build a different architecture, trying to optimize the electrostatic coupling to the DE strips. Therefore, we charged the FEP foil directly in a curved geometry mounted on an auxiliary Stratasys TPU support that allowed us to match the elastomer motion to the electret and to achieve electrical coupling, with no further manipulation after the electret charging realization. In Figure 5a,b, we report the overall scheme and the realized mockup image.

The TPU support (Figure A4) was fabricated with the desired size and shape (5 cm × 4 cm, 0.2 cm thick and 1 cm high in the central part) using the FDM 3D printer F170. In Figure 5a, the black part represents the TPU printed structure, the red layer represents the FEP electret, and the blue layer represents the DE strip. We underline that, if the plane electret is handled to be attached to the TPU support after corona charging, it should lose an important portion of its charge density. To avoid this drawback, the electret is processed by corona charging in the curved geometry directly, after being fixed to the TPU arc supported by a double-sided adhesive.

During the test, in this configuration, the arc support carrying the electret (5 cm × 4 cm) is manually pressed on its middle point, and then returns to the initial position, thanks also to the elastomer flexibility. The EH strips are fixed to the structure on both edges with tape (Figure 5b). As in the previous configuration, the DE strip is facing the negatively charged surface of the electret; its size is 4 cm × 4 cm and thickness of about 30 µm and it holds a Au electrode on the opposite side to the one facing the electret. In Table 2, we summarize the typical parameter values during the test.

#### 2.3.4. Energy Harvesting Tests in Optimized Configuration on Capacitive Load

**Figure 6:**
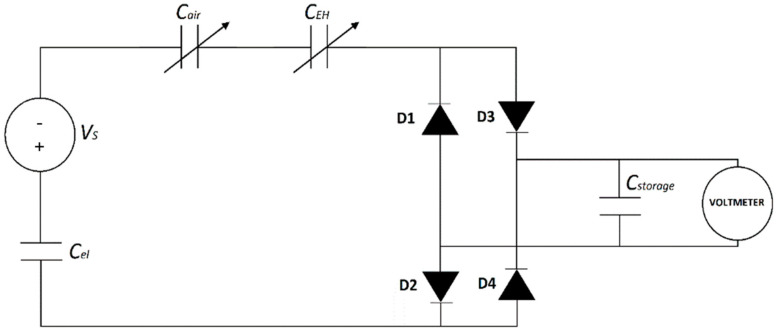
Equivalent circuit of the electret coupling to the EH system with the measurement instruments adopted in the EH in the capacitive load test.

We carried out a test of the accumulation of the device output delivered energy adopting the same architecture described in the previous section. The accumulation circuit was designed to replace the resistive load with a diode bridge carrying the generated charges to a storage capacitor (*C_storage_*) (Figure 6). Here, the goal is to store electrical energy in the *C_storage_*. The diode bridge rectifies the electrical signal, ensuring that some charges are delivered to the capacitor at each half-cycle. The voltage on the capacitor *V_C_* was monitored using the same isolation op-Amp cited above. The instantaneous amount of energy accumulated in the capacitor is dependent on the voltage drop across it through the relation

(5) $$E_{stored}(t)=\frac{1}{2}\;C_{storage}\;V_{C\;}^{2}(t),$$

Experimentally, two different accumulation tests were carried out in the configuration described. The voltage on the storage capacitor was first monitored with the harvester at rest, to accumulate the system leakage current and electromagnetic rubbish only, and secondly with the harvester in periodic motion at 0.5 Hz frequency (see Table 3 for the experimental parameter values).

## 3. Results

### 3.1. Electret Fabrication and Discharge Monitoring

We realized several electrets with the process presented in Section 2.1; we observed that electrets, just after the end of the charging process, show a *V_s_* value that typically spans from −1 to −3 kV, with σ_S_ from −35 to −106 nC/cm^2^.

**Figure 7:**
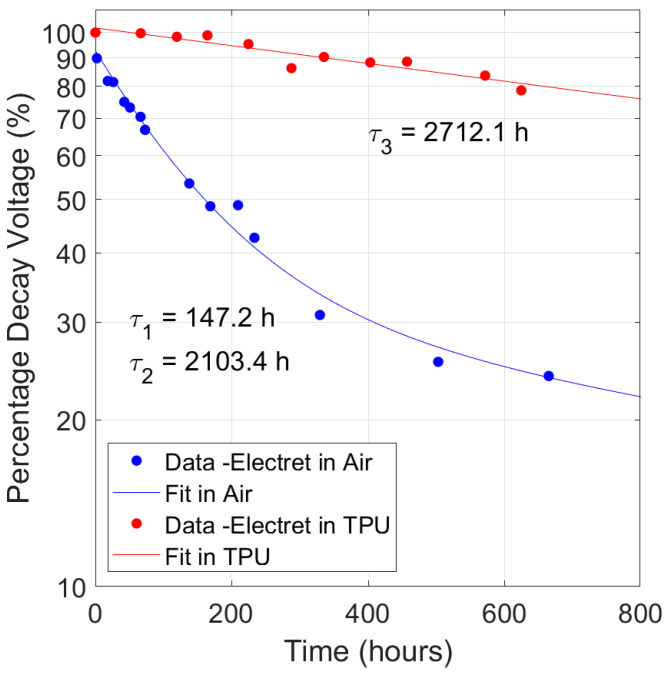
Percentage decay voltage as a function of the time for an electret maintained in air (blue markers) and the same stored in TPU (red markers). For the exponential best fit (red and blue curves), see the main text.

**Figure A3:**
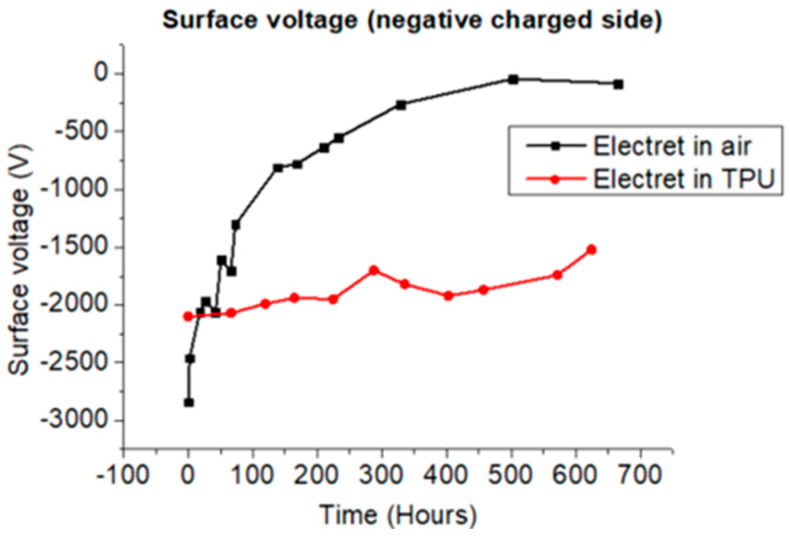
Surface voltage of the negative charged side as a function of time.

**Figure A4:**
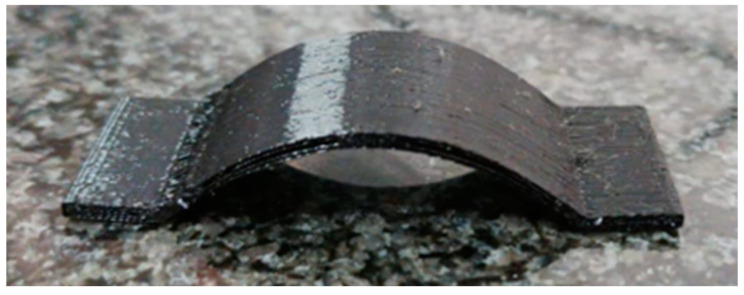
TPU arc support.

Just after their realization, we started to monitor the electrets performances by measuring their surface voltage *V_s_* over time, assessing the stability of the implanted charge and keeping them stored in two different conditions for about 30 days. We monitored their surface voltage percentage decrease as a function of time lapse from realization (Figure 7 and Figure A3). We assumed the same discharge process in both air and TPU ambient, then we adopted the same exponential model for both cases. In Figure 7, the best-fit curves to the percentage decay voltage for both configurations are shown. The discharge process in the air (blue markers) is well adapted to a bi-exponential function $Ae^{-(\frac{t}{\tau_{1}})}+Be^{-(\frac{t}{\tau_{2}})},\;$ with τ_1_ and τ_2_ the time constants of the discharge process. The best-fit time constant values are about 147 h and 2100 h, respectively. The greater value of the duration is linked to the depth of injection of the ions during the corona charging process which creates the so-called volumetric or bulk charges, while the superficial ones are more volatile being less bonded.

On the other hand, the electret stored in the TPU case (6.5 cm × 7.5 cm × 1.2 cm and the walls 0.2 cm thick) shows a slower decaying trend (red markers). A single exponential function ($Ce^{-(\frac{t}{\tau_{3}})}$) fit to the red dotted data gives a time constant value (τ_3_) of about 2700 h. The lifetime of the electrets is clearly higher when they operate surrounded by TPU rather than immersed in air. By comparing the time interval (about 20 h vs. 600 h) in the two sets of experimental data corresponding to the same percentage decay (80%) it is possible to estimate a factor of 20 difference in surface voltage lifetime.

### 3.2. Energy Harvesting Tests

**Figure 8:**
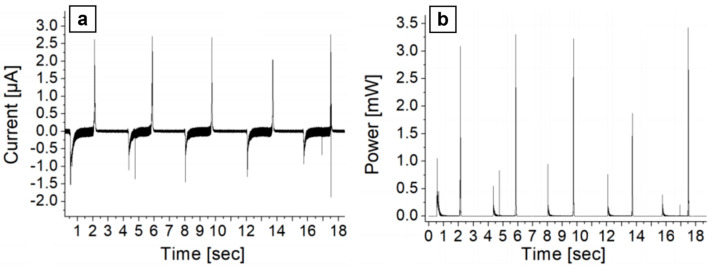
(**a**) Experimental current intensity on *R_load_* vs. time; (**b**) computed power (as explained in the text) vs. time.

In the EH accumulation tests, performed following the procedures in Section 2.2, each energy conversion cycle (elongation-release) produces two opposite polarity peaks on the load resistor with respect to the bare leakage current level (Figure 8a). From this current signal, the power value on the load resistor was then calculated (Figure 8b). Finally, by numerically integrating the power peaks, the energy generated value over a given time interval is obtained; we typically consider the experimental energy value over some tens of cycles (sum over negative and positive peaks) and then derive the figure of merit of average energy generated per cycle and per unit area of EH material.

**Figure 9:**
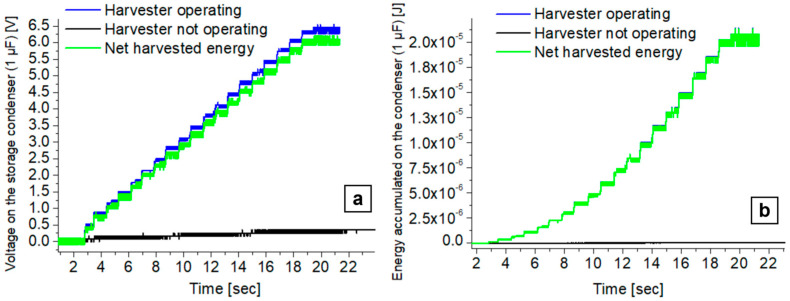
(**a**) Voltage on the storage condenser; (**b**) computed energy on the storage condenser.

In the optimized configuration, we obtained the average energy stored per cycle per unit area of the EH material on the capacitive load by monitoring the voltage V_C_ on the capacitor. For each energy conversion cycle, we obtain two increase steps in V_C_ (Figure 9a,b).

#### 3.2.1. Energy Harvesting Tests on Resistive Load

As reported in Table 4, performing the EH test on resistive load, we observed a mean dissipation energy on the load resistor of 8.6 µJ/cycle (0.6 µJ/cm^2^).

#### 3.2.2. Energy Harvesting Tests in Optimized Configuration on Resistive Load

In the experimental data (Figure 8a,b), the positive peak is often larger than the negative one. We attribute this effect to physical factors related to mechanical tests: (i) elastic properties of the system, concerning the Young modulus of the EH electrostrictive material; (ii) during compression/extension cycles of the arc system of Figure 5b the compression speed—both automated and manual—is higher than the extension speed while returning to the initial rest size.

When optimizing the harvester architecture, the average energy delivered increases to 62 µJ/cycle (3.06 µJ/cm^2^).

#### 3.2.3. Energy Harvesting Tests in Optimized Configuration on Capacitive Load

In Figure 9a, three recorded voltage signals are shown, while in Figure 9b, the corresponding calculated stored energy curve is reported. The green curve represents the net harvested energy, that is the difference between the energy accumulated during motion with respect to the initial rest. In about 20 s of operation, a voltage of about 6 V was reached across the 1 µF capacitor, corresponding to an accumulated energy of 20 µJ. By considering elongation-release cycles at a frequency of 0.5 Hz, this means that each conversion cycle has delivered to the capacitor about 2 µJ (0.1 µJ/cm^2^) of energy. These results are useful figures of merit that allow us to optimize the overall EH device in the end-user configuration.

In Table 4, a summary of all the EH accumulation tests is reported. The “Surface area” and the “Thickness” columns report the electret and the DE geometric characteristics. All these tests were carried out with different electrets, each of them just after the corona charging process with *V_s_* = −1500 V, *σ_S_* = −53 nC/cm^2^. The “Load” column reports the adopted load in that specific test. The “Energy/cycle” and the “Energy/cycle/area” columns report the figure of merit of harvested energy on the load.

In Table 5, the comparison in terms of generated energy and power values is reported, with respect to the literature in references.

## 4. Discussion

In this work, we report on the fabrication and long-term charge retention properties of electrets, made by the corona charging method, and their usage as a bias voltage source for energy harvesting processes coupled with a dielectric elastomer. It is demonstrated that storing electrets in a TPU structure effectively prevents rapid discharge, making this configuration compatible with shoe sole designs and capable of harvesting energy from human gait for potential EH device integration. However, even if the same fabrication method was followed, we observed a *V_s_* fluctuation in the range −3 to −1 kV, corresponding to *σ_S_* from −35 to −106 nC/cm^2^. Indeed, this method could be improved to obtain more predictable results. Two different measurement configurations were investigated. In the first one, an automated slide was used to apply motion duty cycles to the electret plus DEG system. In the second one, a TPU support obtained with a 3D printer was used to maintain the electret bowed and a manual deformation applied—equivalent to pedestrian—a 0.5 Hz deformation.

Through output power delivery tests performed on a load resistor, an energy output of 8.6 µJ/cycle (0.6 µJ/cm^2^) was achieved in a preliminary configuration. In an optimized configuration, on the load resistor, we measured values of 62 µJ/cycle (3.1 µJ/cm^2^). The harvested energy is lower in the first configuration, likely because manipulating the electret after the corona charging process interferes with the charge deposited on it. Therefore, fabricating the electret directly in the curved geometry offers a way to improve energy harvesting performance. This result is significant for our energy harvesting application, which is intended to harvest energy from human gait and be smoothly integrated into a shoe sole structure.

An energy storage test on a capacitor employing a diode bridge was also carried out, succeeding in storing 2 µJ/cycle (0.1 µJ/cm^2^). The harvested energy is lower in this configuration, which highlights a limitation that needs to be addressed. Future studies will focus on optimizing energy storage strategies. In more detail, priority will be given to investigating advanced electronic devices to achieve more efficient energy storage.

The novelty of our approach lies in the direct integration of the DEG with a TPU bridge, ensuring capacitive coupling and enabling soft deformation that adapts to the shoe’s movement during walking. This design enhances energy generation performance, mechanical flexibility, and gait compatibility while maintaining cost-effectiveness. By effectively integrating energy harvesting technology into wearable devices without compromising comfort or flexibility, our system represents a promising solution for wearable energy harvesting applications, offering a practical and efficient means of harnessing biomechanical energy from human motion.

## Figures and Tables

**Table 1:** EH tests on resistive load parameters.

Surface Area (cm^2^)		Thickness (μm)		*R_load_* (MΩ)	Electret *V_s_* (Volt)	Electret *σ_S_* (nC/cm^2^)
Electret	DE ^1^	Electret	DE ^1^			
14 (7 × 2)	12 (6 × 2)	50	50	450	−1500	−53

^1^ Dielectric Elastomer, when not stretched.

**Table 2:** EH tests in optimized configuration on resistive load parameters.

Surface Area (cm^2^)		Thickness (μm)		*R_load_* (MΩ)	Electret *V_s_* (Volt)	Electret *σ_S_* (nC/cm^2^)
Electret	DE ^1^	Electret	DE ^1^			
20 (5 × 4)	16 (4 × 4)	50	30	450	−1500	−53

^1^ Dielectric elastomer, when not stretched.

**Table 3:** EH tests in optimized configuration on capacitive load parameters.

Surface Area (cm^2^)		Thickness (μm)		*C_load_* (μF)	Electret *V_s_* (Volt)	Electret *σ_S_* (nC/cm^2^)
Electret	DE ^1^	Electret	DE ^1^			
20 (5 × 4)	16 (4 × 4)	50	30	1	−1500	−53

^1^ Dielectric elastomer, when not stretched.

**Table 4:** Summary of the EH tests parameters and harvested energies. Taking into account the variability of the material and the production processing, we observed an uncertainty of the energy values of about 20%.

Test ^1^	Surface Area (cm^2^)		Thickness (μm)		Load		Energy/Cycle (µJ)	Energy/Cycle/Area (µJ/cm^2^)
	Electret	DE ^2^	Electret	DE	R_load_ (MΩ)	R_load_ (μF)		
EH on resistive load	14	12	50	50	450	-	8.6	0.6
Optimized configuration on resistive load	20 (5 × 4)	16 (4 × 4)	50	30	450	-	62	3.1
Optimized configuration on capacitive load	20 (5 × 4)	16 (4 × 4)	50	30	-	1	2	0.1

^1^ All these tests are carried out with an electret showing V_s_ = −1500 V, σ_S_ = −53 nC/cm^2^. ^2^ Dielectric elastomer, when not stretched.

**Table 5:** Results comparison with literature.

Ref	Electret *V_s_* (Volt)	Energy/Cycle (µJ)	Peak Power (mW)
Jean-Mistral et al. [5]	−1000	33	-
Lee et al. [7]	−1000	495	-
Genter et al. [8]	−600	-	-
Humming et al. [9]	−550	4.66	-
Huayang Li et al. [10]	−2400	-	54
Peter et al. [11]	−500	206	-
Sakane et al. [12]	−640	-	0.7
**This work**	−1500	62	3

## Data Availability

The original contributions presented in this study are included in the article/Supplementary Material. Further inquiries can be directed to the corresponding author.

## Supplementary Materials

Supplementary materials related to this article can be found online at: https://fitnessmaterialiavanzati2019.wordpress.com/news accessed on 30 January 2025.

## Abbreviations

The following abbreviations are used in this manuscript:

DE	Dielectric elastomer
DEG	Dielectric elastomer generator
EH	Energy harvesting
TPU	Thermoplastic polyurethane
PTFE	Polytetrafluoroethylene
THV	Tetrafluorethylene-hexafluorpropylene vinylidenfluoride
PDMS	Polydimethysiloxane
FEP	Fluorinated Ethylene propylene
PET	Polyethylene terephthalate
HV	High voltage
CCTO	CaCu_2_Ti_4_O_12_
FDM	Fused deposition modeling
